# A Specific for Scabies

**Published:** 1858-12

**Authors:** 


A SPECIFIC FOR SCABIES.

At the last meeting of the Academy of Science, Paris, M. Bonnet, of Epinal, sent in a paper announcing that benzine is a specific for the itch. The author of the paper states that if benzine be rubbed on the parts affected, and also very slightly on the other parts of the body, a cure will be effected in the course of five minutes, after which the patient may take a warm bath for half an hour. Nevertheless, in cases where the itch is accompanied with secondary eruption, the latter will require a separate treatment.—*London Lancet.*

